# Blue light exposure-dependent improvement in robustness of circadian rest-activity rhythm in aged rats

**DOI:** 10.1371/journal.pone.0292342

**Published:** 2023-10-04

**Authors:** Eryck Holmes A. Silva, Nelyane Nayara M. Santana, Narita Renata M. Seixas, Lyzandro Lucas F. Bezerra, Maria Milena O. Silva, Sâmarah F. Santos, Jeferson S. Cavalcante, Mário A. Leocadio-Miguel, Rovena Clara Engelberth

**Affiliations:** 1 Laboratory of Neurochemical Studies, Department of Physiology and Behavior, Biosciences Center, Federal University of Rio Grande do Norte, Natal, Brazil; 2 International Institute Edmond and Lily Safra, Santos Dumont Institute, Macaíba, Brazil; 3 Department of Psychology, Northumbria University, Newcastle upon Tyne, United Kingdom; Meiji University, School of Agriculture, JAPAN

## Abstract

The aging effects on circadian rhythms have diverse implications including changes in the pattern of rhythmic expressions, such as a wide fragmentation of the rhythm of rest-activity and decrease in amplitude of activity regulated by the suprachiasmatic nucleus (SCN). The study of blue light on biological aspects has received great current interest due, among some aspects, to its positive effects on psychiatric disorders in humans. This study aims to evaluate the effect of blue light therapy on the SCN functional aspects, through the evaluation of the rest-activity rhythm, in aging rats. For this, 33 sixteen-months-old male Wistar rats underwent continuous records of locomotor activity and were exposed to periods of 6 hours of blue light during the first half of the light phase (Zeitgeber times 0–6) for 14 days. After this, the rats were maintained at 12h:12h light:dark cycle to check the long-term effect of blue light for 14 days. Blue light repeated exposure showed positive effects on the rhythmic variables of locomotor activity in aged rats, particularly the increase in amplitude, elevation of rhythmic robustness, phase advance in acrophase, and greater consolidation of the resting phase. This effect depends on the presence of daily blue light exposure. In conclusion, our results indicate that blue light is a reliable therapy to reduce circadian dysfunctions in aged rats, but other studies assessing how blue light modulates the neural components to modulate this response are still needed.

## 1 Introduction

Aging is the process that takes place in structures, systems, and organisms with the passage of time [1, 2]. Whereas senescence is the process accompanied by a time-dependent physiological weakening [3], senility relates to degeneration processes arising from accessory comorbidities throughout senescence [4]. Although aging cannot be characterized as a process of pathological origin, it is a physiological process that generates greater vulnerability to existing pathologies [5, 6]. Thus, it affects most living organisms and emerges at the molecular, cellular, systemic, and organic levels due to genetic, epigenetic, and environmental modulatory actions [7, 8]. In general, aging is explained by two groups of theories: those that say it is a programmed process, and those that say it is caused by damages and/or errors to the organism functioning [9–11].

Potential elucidations of how aging affects the circadian timing system (CTS) include observations that it is involved in metabolic regulatory processes, such as regulation of reactive oxygen species [12], melatonin release [13], DNA repair system [14], autophagy activities [15] and others [8]. When these processes are dysregulated, the potential increase in oxidative stress and the tendency to form protein aggregates lead to neurodegeneration and decreased cognitive function, phenomena related to aging [16]. In this way, CTS can work to delay senescence whilst aging negatively affects CTS, and thus the senescence processes escalate [2, 17]. The CTS presents phases of formation, maturation, and senescence [18]. Major changes in biological rhythms occur in the early and late stages of life [19]. Thus, while the formation and consolidation of temporal organization occur at the beginning of ontogenesis, the fragmentation and loss of rhythmicity occur at the end of life [19].

Changes in the neural components of the suprachiasmatic nucleus (SCN), an endogenous master clock of the hypothalamus in the mammalian brains, and in the circadian rhythmic expression pattern in mammals have been associated with increasing age [20, 21]. These changes include a fragmentation of the rest-activity rhythm [22], difficulty in resynchronization [23], a decrease in amplitude [24], and shortening of the circadian period [25], resulting in a modified response to both photic and non-photic stimuli [26, 27]. In humans, aging is associated with reduced sleep duration at night, reduction of the proportion of deep sleep stage, and increase in the number of naps during the day, representing a decrease in the amplitude and an increase in the fragmentation of the rest-activity rhythm [28, 29].

Currently, among the non-pharmacological treatments suggested to play role on the aged CTS, the increase in activity levels or exposure to light to promote the regularity of biological rhythms stands out [30, 31]. The cyclic variation of light strongly synchronizes and coordinates the temporal rhythms of physiology and behavior by sending signals to multiple structures in the brain including the central circadian clock [32, 33]. The positive effects of light on the CTS are determined by several factors, including intensity [34, 35], wavelength [36], duration [37], and the prior photic history [38, 39]. Light is used as a practical treatment in several fields of medicine, for example, to minimize circadian rhythm sleep–wake disorders in shift workers [40–43]. Studies on people with seasonal affective disorder (SAD) have shown that light administered at certain times of the day can correct a possible change in the phase of central oscillator-controlled melatonin release, with significant improvement in mood [44, 45].

Light crosses the retinal circuit through two functional pathways: the visual, transmitting retinal signals directly to the visual cortex, and the non-visual associated with photic information in the modulation of physiological and behavioral processes to hypothalamic retinorecipient centers, such as the SCN [46, 47]. More recent studies have focused on the use of monochromatic blue light, which is considered effective in improving mood in human models of cognitive alterations [48, 49]. Blue light promotes a direct stimulus to melanopsin, a protein that acts as a photoreceptor within the subset of intrinsically photosensitive retinal ganglion cells (ipRGCs) [32, 34]. The signal generated by ipRGCs (M1 and M2 type cells) is conducted through the retinohypothalamic tract to SCN neurons in the hypothalamus [50]. The ipRGCs containing melanopsin are strongly stimulated by monochromatic blue light (∼480 nm range) and minimally influenced by polychromatic white light (∼450 nm range), which needs a higher intensity to actually promote a functional change in the SCN and stimulate the ipRGCs [51]. Growing evidence supports the view that the effects of blue light on human sleep and brain activity during wakefulness, as well as sleep duration and the homeostatic response to sleep loss, depend on both melanopsin and circadian timing [32].

Considering the gap in parameter standardization found in clinical trials testing blue light in humans (light intensity, period, and time of application), the mechanisms underlying the behavioral improvement are still unknown. Blue light treatment in aging disorders is described only in its self-evaluative behavioral aspects by the investigated subjects [52]. Few studies bring the effectiveness of phototherapy and do not reveal several aspects that are essential for the continued use of this technique in humans. To answer these questions, rats were used as behavioral models, given their similarity with pathophysiological processes that occur in humans, such as neurodegeneration and circadian dysregulation [53]. This study evaluated the effects of blue light therapy on the rest-activity rhythm in aged rats. We hypothesized that blue light exposure attenuates circadian dysfunction in aging by increasing rhythm robustness and decreasing rhythm fragmentation.

## 2 Material and methods

### 2.1 Animals

A total of 33 sixteen-months-old male Wistar rats (650-690g) were individually housed in polypropylene cages (40 x 32 x 17 cm) at soundproof room with controlled temperature (24 ± 1°C) and humidity (50%), on a 12h:12h light:dark cycle (white lamps; 350 lux during photophase; 2 lux during scotophase; lights on at 6:00 am–Zeitgeber time, ZT 0 and lights off at 18:00 pm–ZT 12) with food (rodent chow, Labina and Purina; 3.5 kcal/g) and water freely available. Additionally, 12 young rats (3 months old) were used to evaluate the ontogenetic effect on the circadian rhythmicity pattern and the blue light exposure effect on youthful circadian rhythm. All animals were maintained undisturbed except for two weekly bedding changes and food/water replenishment to ensure ad libitum regimen (between ZT12 –ZT18). All efforts were made to minimize animal pain, suffering, or discomfort in all experimental conditions. All procedures were approved by the local ethics committee (CEUA-UFRN number 146.074/2018) in accordance with Brazilian law number 11.794/2008 for animal experimentation.

### 2.2 Protocol of blue light-exposure and housing conditions

Light-tight chambers controlled photic conditions defined by polychromatic white and monochromatic blue lamps. Illumination was measured using a digital photometer (MLM-1011 Minipa). Wavelength emitted by the lamps was measured by a spectrometer (Fiber Optic Spectrometer USB4000 UV-VIS, Ocean Optics, Inc. USA) using the Spectra Suite software (Ocean Optics, Inc. USA). The blue lamps were vertically positioned on the top of every single cage at the same height as the white lamps providing a total ~500 lux. The measurements were made without the cages in the chamber and thus represent the maximum irradiance to which the rats could have been exposed.

**Fig 1:**
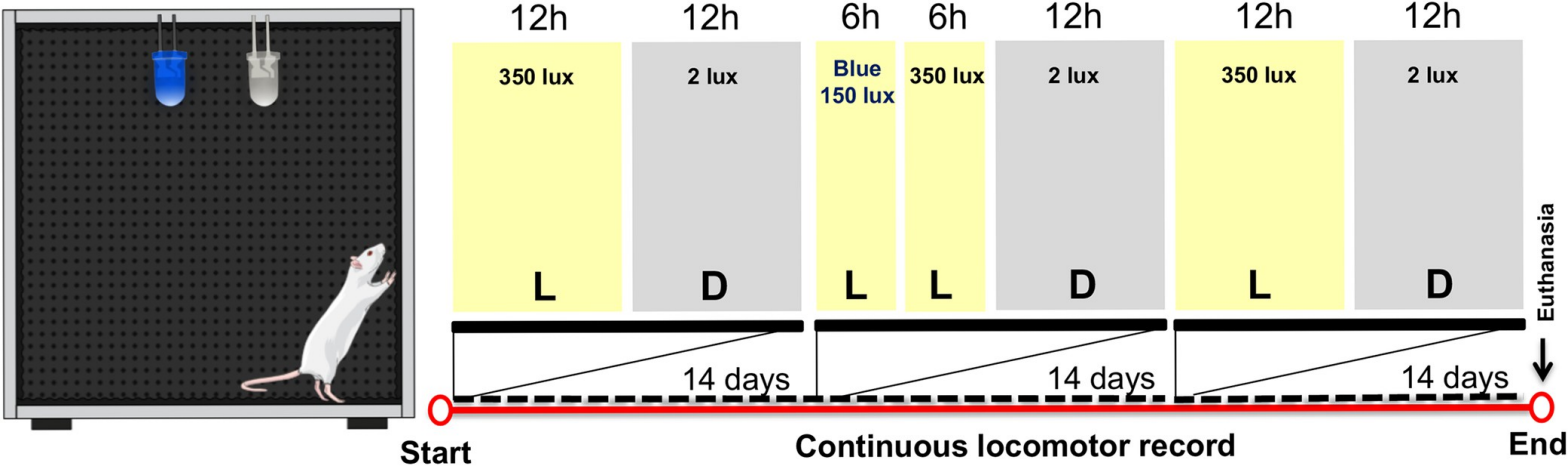
Diagrammatic representation of the blue light therapy (6+6h:12h blue+white light:dark) and housing conditions (12h:12h light:dark). Yellow indicates the light phase (L); gray indicates the dark phase (D).

Simultaneously, aged animals were habituated in the experimental room and synchronized to the light:dark cycle (350:2 lux; peak at 450–470 nm) for 14 days before the blue light therapy. Behavioral baseline measurements were collected and then it was applied an additional exposure to blue light (150 lux; peak at 470–490 nm) during the first half of the light phase (ZT 0–6) for 14 days. After that, the animals were again submitted to the light:dark cycle (350:2 lux; peak at 450–470 nm) to check the long-term effect of blue light for additional 14 days prior to euthanasia. Each animal’s locomotor activity was recorded continuously during the light treatment (see Fig 1 for details).

The young control group was subjected simultaneously to the same light:dark cycle (350:2 lux; peak at 450–470 nm) for 14 days to obtain the young locomotor activity pattern. And later, they received an additional exposure to blue light (150 lux; peak at 470–490 nm) during the first half of the light phase (ZT 0–6) for 14 days, totaling twenty-eight experimental days. The purpose of inserting the group of young animals is to demonstrate the ontogenetic effect on the robustness of the circadian rhythm, as well as to investigate the influence of exposure to blue light on the young circadian rest-activity rhythm.

### 2.3 Assessment of rest-activity rhythm

The circadian rest-activity rhythm was measured by assessing the animal’s spontaneous locomotor pattern over the whole experimental period. For that, locomotor activity was monitored using passive infrared motion captors placed 15 cm away and above each animal’s cage. Data was processed with a computerized data acquisition system (National Instruments PCI-6025E) driven by SAP software (data acquisition system and software developed by Rodrigues and Araújo, Laboratory of Neurobiology and Biological Rhythms–UFRN, 2012). Data were continuously acquired and stored. Locomotor activity was expressed as 5-minute time bins (totalling 288 recordings over 24 hours).

To determine the robustness of circadian rhythmicity, the cosinor method and non-parametric variables were used in El temps software (developed by Dr. Antoni Díez-Noguera, University of Barcelona, 1999). First, to visualize the dominant pattern of rest-activity rhythm in each segment of the experiment (i.e., Basal LD, Blue light LD, and Post-blue light LD) actograms and mean activity curves (with % area under curve—%AUC) were used [54]. Secondly, a cosinor curve fitting technique was performed to determine the following rhythmic parameters: rhythm-adjusted mean (midline estimating statistic of rhythm—MESOR), acrophase, and amplitude of the fitted curve [55, 56]. Thus, the technique used analyzed the locomotor activity through a periodic regression that facilitated the visualization and analysis of the confidence intervals of these estimated parameters, leading to a classic cosinor method [57]. Then the total locomotor activity was evaluated for the entire day (total activity per 24 hours), at the start of activity phase (ZT), and at the end of activity phase (ZT) [58, 59]. The procedure for identifying the start and end of activity phase was performed through a regression analysis between the data and the Heaviside function (the unit step function) as previously described [57]. Activity levels with the infrared sensors are reported as counts. Finally, to better understand our data pattern, nonparametric analysis was performed, using the variables interdaily stability (IS), intradaily variability (IV), least active five-hour period (L5), and most active ten-hour period (M10) [60–62]. IS quantifies the strength of resemblance from 1 day to the next (i.e. robustness); and IV measures the fragmentation between rest-activity.

### 2.4 Statistical analysis

A Kolmogorov-Smirnov test was performed to assess the normality of our group distributions. For paired analyses, we used Wilcoxon test and for unpaired analyses, it was used Mann-Whitney test. In all analyses, differences were considered significant at p<0.05 and data are expressed as median ± interquartile range. Data analyses were performed with the software GraphPad Prism version 7 for Microsoft Windows.

## 3 Results

### 3.1 Ontogenetic effects on the pattern of locomotor activity: Young vs aged

All animals were evaluated for the locomotor activity pattern and there was no blue light exposure in this ontogenetic comparison. Statistical analysis revealed that young rats exhibited entrained and robust rest-activity rhythms during baseline recording. In contrast, aged animals showed continuous fragmentation of the rest-activity and, therefore, a rhythm robustness reduction. All data from this comparison can be accessed in detail in Table 1.

**Table 1:** Locomotor activity pattern analysis of young and aged rats entrained to the 12h:12h light:dark cycle for 14 days. Values expressed as median ± interquartile range.

Parameters	Young (3mo)	Aged (16mo)	U-value	p-value
Total daily activity (a.u.)	16640 ± 14982–18298	13010 ± 8714–16003	26	0.0068*
Start of activity phase (ZT)	13.63 ± 12.57–14.19	11.49 ± 11.2–11.76	10	0.0001*
End of activity phase (ZT)	23.23 ± 22.86–23.48	23.11 ± 22.37–23.44	58	0.43
Mesor (a.u.)	57.89 ± 52.12–63.65	43.07 ± 30.19–55.54	25	0.0056*
Amplitude (a.u.)	44.83 ± 42.53–50.56	26.22 ± 22.72–29.59	1	0.0001*
Acrophase (ZT)	19.09 ± 18.39–19.21	16.54 ± 15.91–16.78	0	0.0001*
IS (a.u.)	0.4444 ± 0.4001–0.4806	0.227 ± 0.2008–0.2503	0	0.0001*
IV (a.u.)	0.442 ± 0.4243–0.4763	0.5665 ± 0.5303–0.6243	10	0.0001*
M10 (a.u.)	96.9 ± 87.49–107.3	65 ± 49.18–79.59	6	0.0001*
L5 (a.u.)	13.74 ± 10.6–19	21.23 ± 11.57–26.97	43	0.10

U: Mann-Whitney U test; a.u., arbitrary unit; ZT, zeitgeber time; IS, interdaily stability; M10, most active ten-hour period; L5, least active five-hour period; IV, intradaily variability

*significant p-value.

### 3.2 Blue light effects in young circadian rhythm

**Fig 2:**
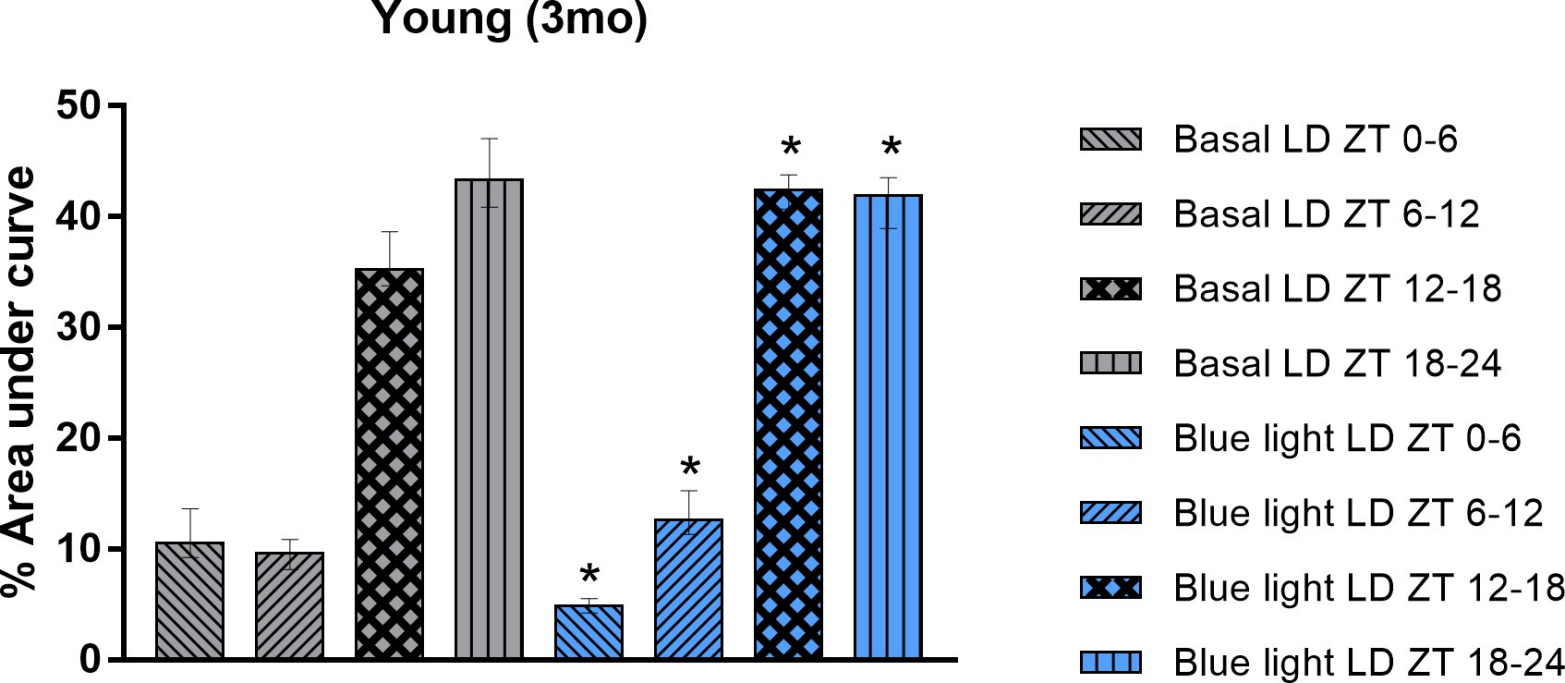
Locomotor activity pattern of young rats submitted to the Basal LD (12h:12h light:dark) and Blue light LD (6+6h:12h blue+white light:dark) conditions at different times of the cycle. Descriptive graph of the % area under curve (%AUC) of young rats (n = 12) locomotor activity submitted to the Basal LD (for 14 days) and Blue light LD (for 14 days) conditions. Values expressed as median ± interquartile range. Drawings of each column represents the range selected to represent part of a cycle. Wilcoxon test, p-value: p<0.05; * indicates significant difference compared to Basal LD; ZT, zeitgeber time; LD, light:dark.

All treated young animals followed the same experimental photic conditions proposed for aged animals (See topic 2.2 for details). First, we closely examined the locomotor activity patterns of young rats at different times of the day. Specifically, we observed their activity during the first six hours of the daytime (ZT 0–6), the latter six hours of the daytime (ZT 6–12), the first six hours of the nighttime (ZT 12–18), and the last six hours of the nighttime (ZT 18–24). Our findings revealed that the young rats were significantly more active during the dark phase in both experimental stages (Basal LD and Blue light LD), as shown in Fig 2. Statistical analysis revealed that the %AUC decreased at ZT 0–6 between Basal LD (10.56 ± 9.21–13.63) vs Blue light LD (4.9 ± 4.248–5.52; Wilcoxon test, W = -78, p = 0.0005). Differences were reported in ZT 6–12 between Basal LD (9.665 ± 8.145–10.86) vs Blue light LD (12.64 ± 11.32–15.26; Wilcoxon test, W = 76, p = 0.0010) and at ZT 12–18 between Basal LD (35.23 ± 33.79–38.63) vs Blue light LD (42.4 ± 40.7–43.76; Wilcoxon test, W = 78, p = 0.0005), where the %AUC values increased. Finally, the %AUC decreased at ZT 18–24 between Basal LD (43.31 ± 40.84–47.07) vs Blue light LD (41.92 ± 38.93–43.52; Wilcoxon test, W = -58, p = 0.02).

Secondly, young rats exhibited important parameter changes related to circadian robustness after blue light was applied. Statistical analysis revealed that exposure to blue light promoted early activity phase advancement, acrophase advancement, increased circadian robustness, and resting phase consolidation in young rats treated with blue light for 14 days. All data from this analysis can be accessed in detail in Table 2.

**Table 2:** Locomotor activity pattern analysis of young rats (n = 12) submitted to the Basal LD (for 14 days) and Blue light LD (for 14 days) conditions. Values expressed as median ± interquartile range.

Parameters	Young (3mo)		W-value	p-value
	Basal LD	Blue light LD		
Total daily activity (a.u.)	16640 ± 14982–18298	14405 ± 13041–16365	-72	0.0024*
Start of activity phase (ZT)	13.63 ± 12.57–14.19	12 ± 11.4–12.32	-78	0.0005*
End of activity phase (ZT)	23.23 ± 22.86–23.48	23.2 ± 23.05–23.66	18	0.51
Mesor (a.u.)	57.89 ± 52.12–63.65	58.27 ± 54.27–65.28	22	0.42
Amplitude (a.u.)	44.83 ± 42.53–50.56	47.5 ± 43.8–52.33	46	0.07
Acrophase (ZT)	19.09 ± 18.39–19.21	17.43 ± 16.88–17.62	-78	0.0005*
IS (a.u.)	0.4444 ± 0.4001–0.4806	0.4908 ± 0.4688–0.4993	78	0.0005*
IV (a.u.)	0.442 ± 0.4243–0.4763	0.487 ± 0.4383–0.5473	48	0.06
M10 (a.u.)	96.9 ± 87.49–107.3	95.51 ± 87.9–107.6	-32	0.23
L5 (a.u.)	13.74 ± 10.6–19	10.2 ± 8.613–12.35	-72	0.0024*

W: Wilcoxon test; a.u., arbitrary unit; ZT, zeitgeber time; IS, interdaily stability; M10, most active ten-hour period; L5, least active five-hour period; IV, intradaily variability

*significant p-value.

### 3.3 Actograms and 24-hour activity profile in aging

**Fig 3:**
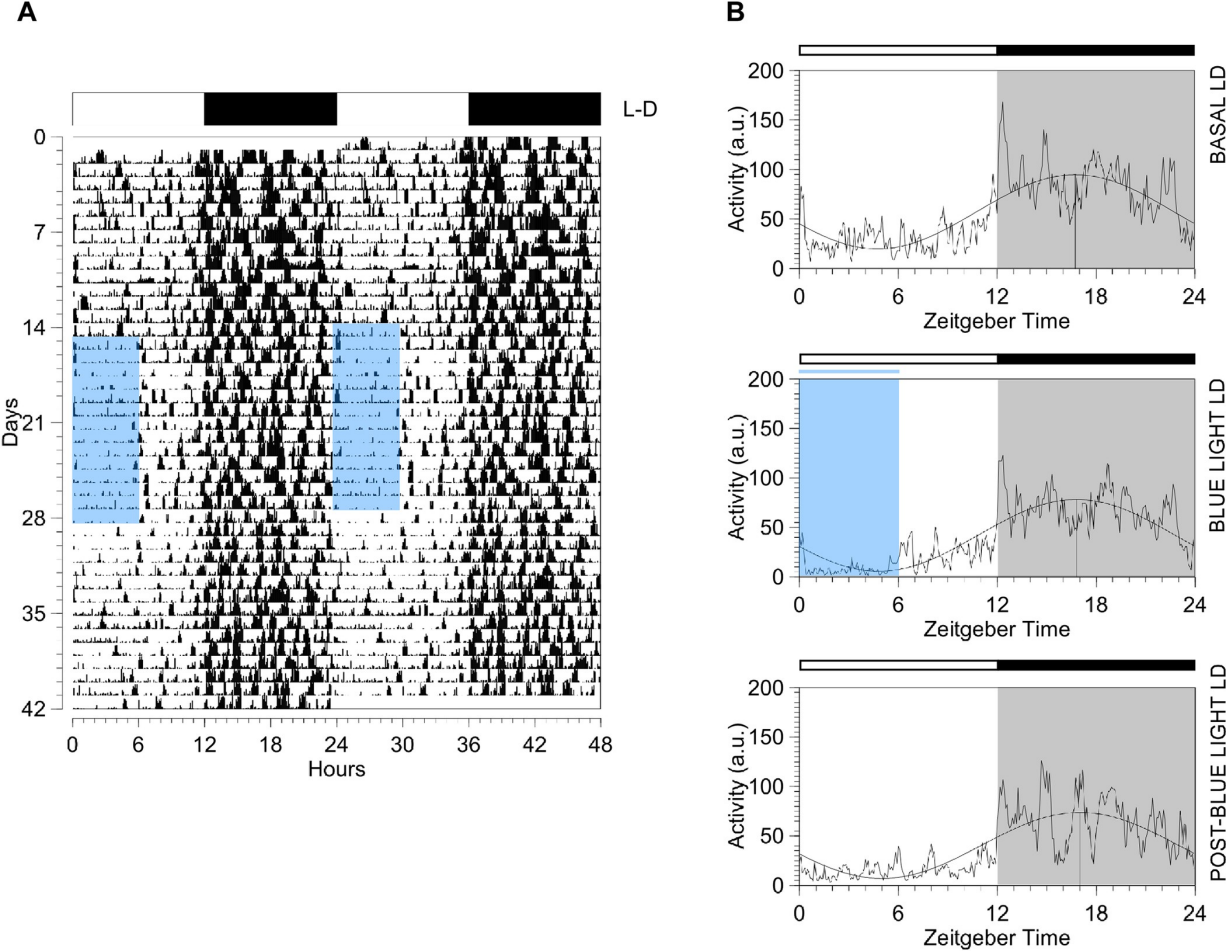
Actogram and mean activity curves showing the locomotor activity rhythm of an aged rat submitted to the Basal LD (12h:12h light:dark), Blue light LD (6+6h:12h blue+white light:dark), and Post-blue light LD (12h:12h light:dark) conditions. (A) Actogram of the activity plotted in 48 h represented on the line and days arranged sequentially from top to bottom. Vertical deflections represent the activity value for each record collected at 5-minute intervals. The white/black bar at the top indicates the 12h:12h light:dark cycle and the blue shading indicates the time of exposure to blue light in relation to hours. Days 1–14: Baseline recording. Days 15–28: Blue light therapy. Days 29–42: Post-blue light therapy. (B) Mean activity curves of aged rat versus hours at Basal LD, Blue light LD, and Post-blue light LD stages. The white/black bar at the top indicates the 12h:12h light:dark cycle. Blue shading indicates the time of exposure to blue light in relation to hours and the gray shading represents the animal’s activity phase. The sine wave represents the rhythm-adjusted mean (Mesor) and the vertical line represents the peak of activity (Acrophase) of the animal. a.u., arbitrary unit; L-D, light:dark cycle.

Typically, rats’ activity pattern over 24 h is characterized by the concentration of activity in the dark phase. The actogram of aged rat showed that the expected pattern was observed, but the locomotor activity changed in response to blue light exposure. From the fifteenth experimental day, animals expressed more activity blocks during the first half of the dark phase (i.e., ZT12 to ZT18; Fig 3A). In addition to the actogram, the profiles of the locomotor activity rhythm in 24 hours, in the average activity curves, seem to show decreased activity block in the light phase, as evidenced in Fig 3B during the first half of the light phase (i.e., ZT0 to ZT6).

**Fig 4:**
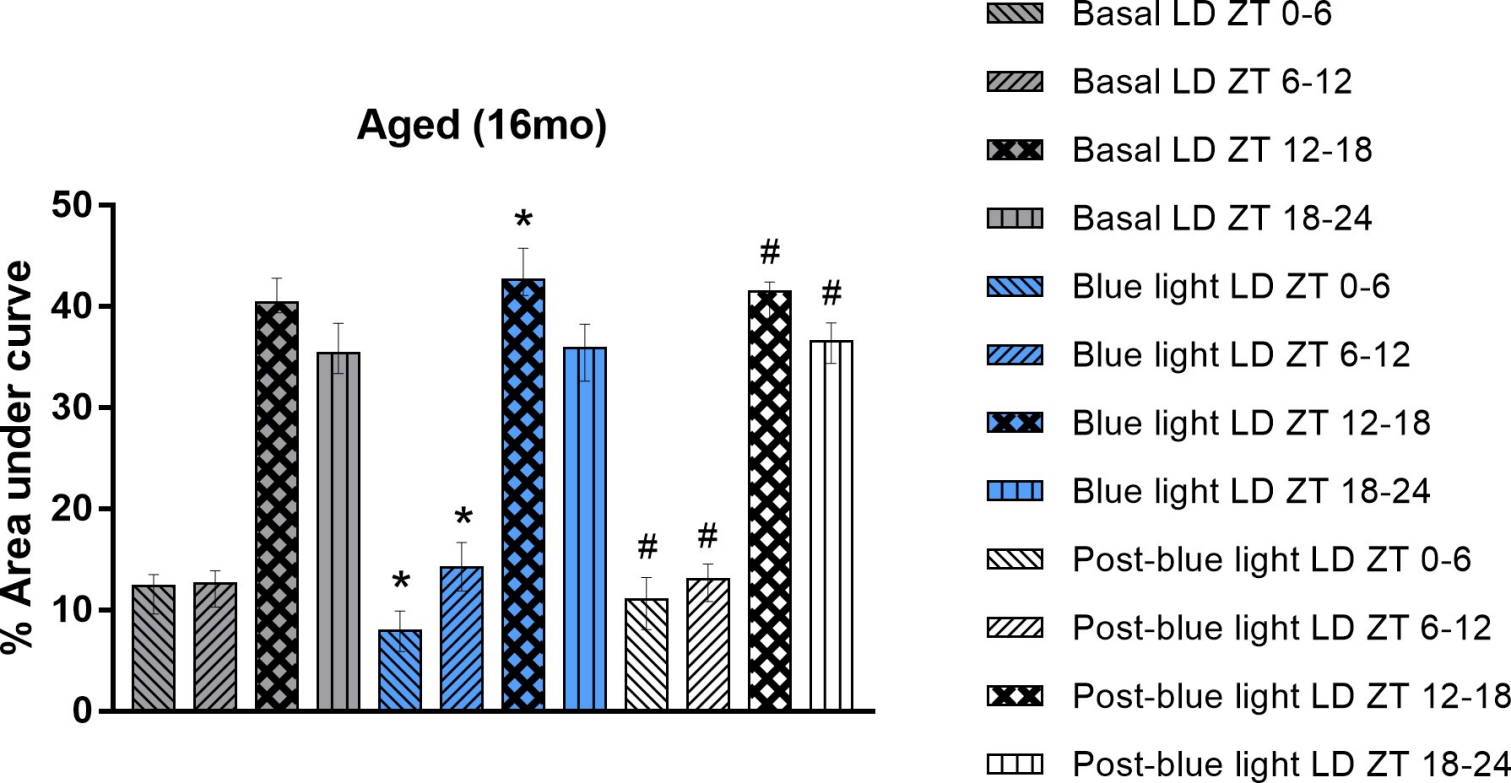
Locomotor activity pattern of aged rats submitted to the Basal LD (12h:12h light:dark), Blue light LD (6+6h:12h blue+white light:dark), and Post-blue light LD (12h:12h light:dark) conditions at different times of the cycle. Descriptive graph of the % area under curve (%AUC) of aged rats (n = 33) locomotor activity submitted to the Basal LD (for 14 days), Blue light LD (for 14 days), and Post-blue light LD (for 14 days) conditions. Values expressed as median ± interquartile range. Drawings of each column represents the range selected to represent part of a cycle. Wilcoxon test, p-value: p<0.05; * indicates significant difference compared to Basal LD; # indicates significant difference compared to Blue Light LD; ZT, zeitgeber time; LD, light:dark.

To confirm the observed 24-hour activity profile, the locomotor activity pattern of aged rats was closely evaluated at different times of the day. Specifically, we observed their activity during the first six hours of the daytime (ZT 0–6), the latter six hours of the daytime (ZT 6–12), the first six hours of the nighttime (ZT 12–18), and the last six hours of the nighttime (ZT 18–24). The activity of aged rats was characterized by the concentration of activity in the dark phase in the three experimental stages (Basal LD, Blue light LD, and Post-blue light LD), as shown in Fig 4. Statistical analysis revealed that the %AUC decreased at ZT 0–6 between Basal LD (12.38 ± 9.6–13.54) vs Blue light LD (7.95 ± 5.875–9.905; Wilcoxon test, W = -543, p<0.0001). However, an increase in %AUC values was reported between Blue light LD vs Post-blue light LD (11.02 ± 8.07–13.22; Wilcoxon test, W = 519, p<0.0001), and there were no relevant differences between Basal LD vs Post-blue light LD (Wilcoxon test, W = -211, p = 0.06).

An increase in %AUC was reported in ZT 6–12 between Basal LD (12.64 ± 10.3–13.92) vs Blue light LD (14.22 ± 11.9–16.7; Wilcoxon test, W = 483, p<0.0001). However, a decrease in %AUC values was reported between Blue light LD vs Post-blue light LD (13.06 ± 10.86–14.58; Wilcoxon test, W = -449, p<0.0001), and there were no relevant differences between Basal LD vs Post-blue light LD (Wilcoxon test, W = 149, p = 0.19). In ZT 12–18, %AUC values increased significantly between Basal LD (40.44 ± 39.39–42.82) vs Blue light LD (42.69 ± 41.1–45.79; Wilcoxon test, W = 411, p = 0.0001). On the other hand, a decrease in %AUC was reported between Blue light LD vs Post-blue light LD (41.46 ± 38.86–42.45; Wilcoxon test, W = -363, p = 0.0008), and there were no relevant differences between Basal LD vs Post-blue light LD (Wilcoxon test, W = -77, p = 0.50). Finally, in ZT 18–24, there were no significant differences in %AUC between Basal LD (35.39 ± 33.41–38.36) vs Blue light LD (35.94 ± 32.64–38.28; Wilcoxon test, W = -85, p = 0.45). When compared Blue light LD vs Post-blue light LD (36.57 ± 34.41–38.4; Wilcoxon test, W = 265, p = 0.016) there was an increase in %AUC values. For Basal LD vs Post-blue light LD, Wilcoxon test did not show statistical significance (W = 189, p = 0.09).

### 3.4 Amplitude, acrophase and total activity in aging

**Fig 5:**
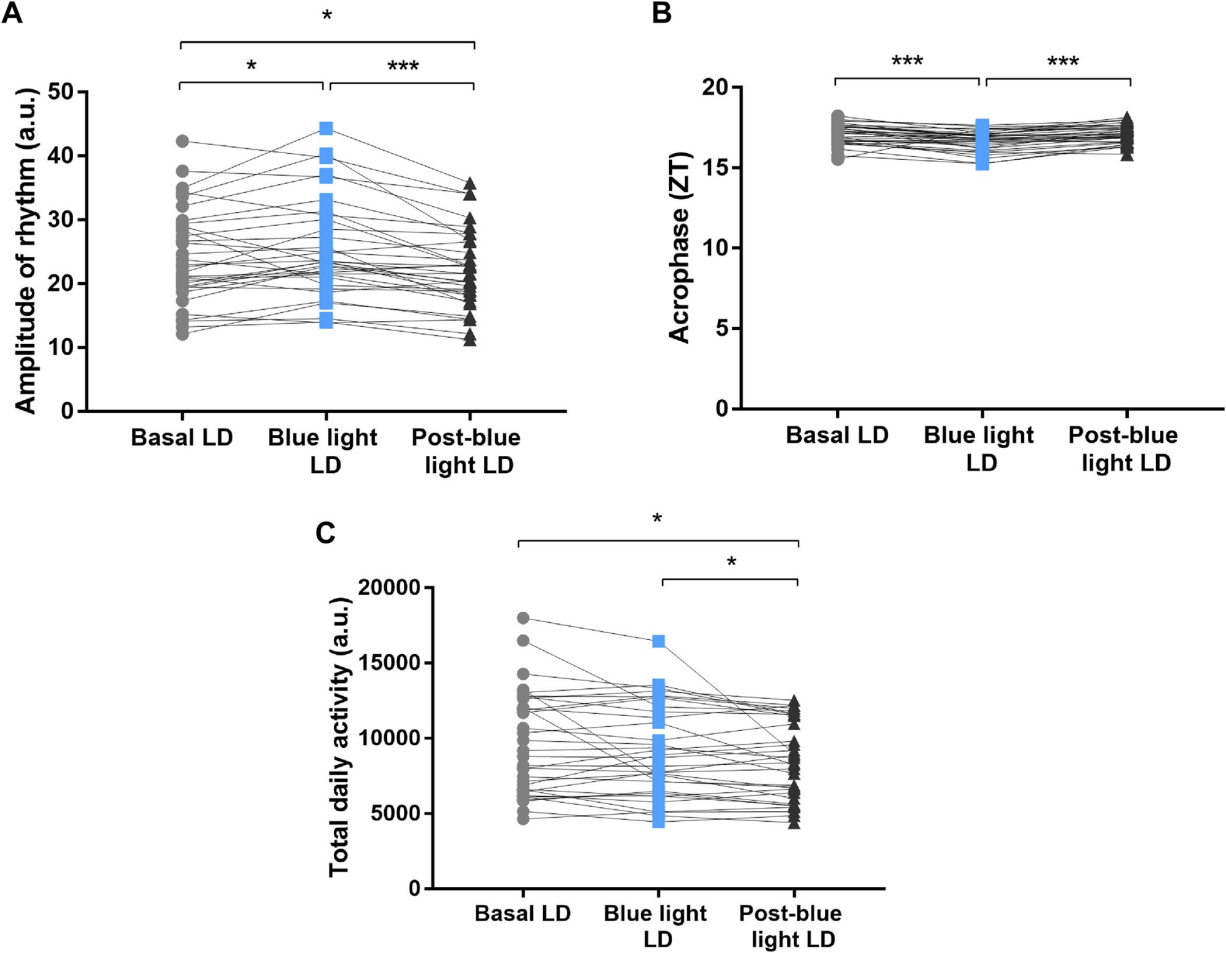
Variables derived from cosinor curve fitting. Descriptive graphs of the amplitude (A), acrophase (B), and total daily activity (C) of aged rats (n = 33) locomotor activity submitted to the Basal LD (for 14 days), Blue light LD (for 14 days), and Post-blue light LD (for 14 days) conditions. Values expressed as median ± interquartile range. Circles, squares, and triangles represent values of each animal evaluated. a.u., arbitrary unit; ZT, zeitgeber time; LD, light:dark; Wilcoxon test, p-value: *p<0.05; ***p<0.001.

The variables derived from cosinor curve fitting of aged rat can be observed in Fig 5. Amplitude refers to the distance from the mesor to the peak or to the trough of the rhythm and is described in arbitrary units. Regarding the rhythm amplitude, significant differences were observed between Basal LD (22.54 ± 19.39–29.15) vs Blue light LD (23.39 ± 20.38–30.4; Wilcoxon test, W = 233, p = 0.03), where the aged animals showed an increase in amplitude values (Fig 5A). Concomitantly, a decrease in rhythm amplitude values was found between Blue light LD vs Post-blue light LD (21.61 ± 18.21–26.66; Wilcoxon test, W = -479, p<0.0001), and between Basal LD vs Post-blue light LD (Wilcoxon test, W = -251, p = 0.02; Fig 5A).

Acrophase, the measure of the time of overall high values recurring in each cycle, was converted to hours (ZT). Regarding acrophase, significant differences were observed between Basal LD (17.2 ± 16.61–17.57) vs Blue light LD (16.81 ± 16.22–17.09; Wilcoxon test, W = -453, p<0.0001), and between Blue light LD vs Post-blue light LD (17.08 ± 16.63–17.54; Wilcoxon test, W = 506, p<0.0001; Fig 5B). For Basal LD vs Post-blue light LD, Wilcoxon test did not show statistical significance (W = -23, p = 0.84; Fig 5B).

Regarding the total daily activity, the aged animals showed no changes in activity when compared to Basal LD (8789 ± 6514–12337) vs Blue light LD (8708 ± 6439–11924; Wilcoxon test, W = -97, p = 0.39). However, a decrease in total activity values was reported between Blue light LD vs Post-blue light LD (8368 ± 6199–11540; Wilcoxon test, W = -227, p = 0.04), and Basal LD vs Post-blue light LD (Wilcoxon test, W = -269, p = 0.01; Fig 5C).

### 3.5 Entrainment and circadian function in aging

**Fig 6:**
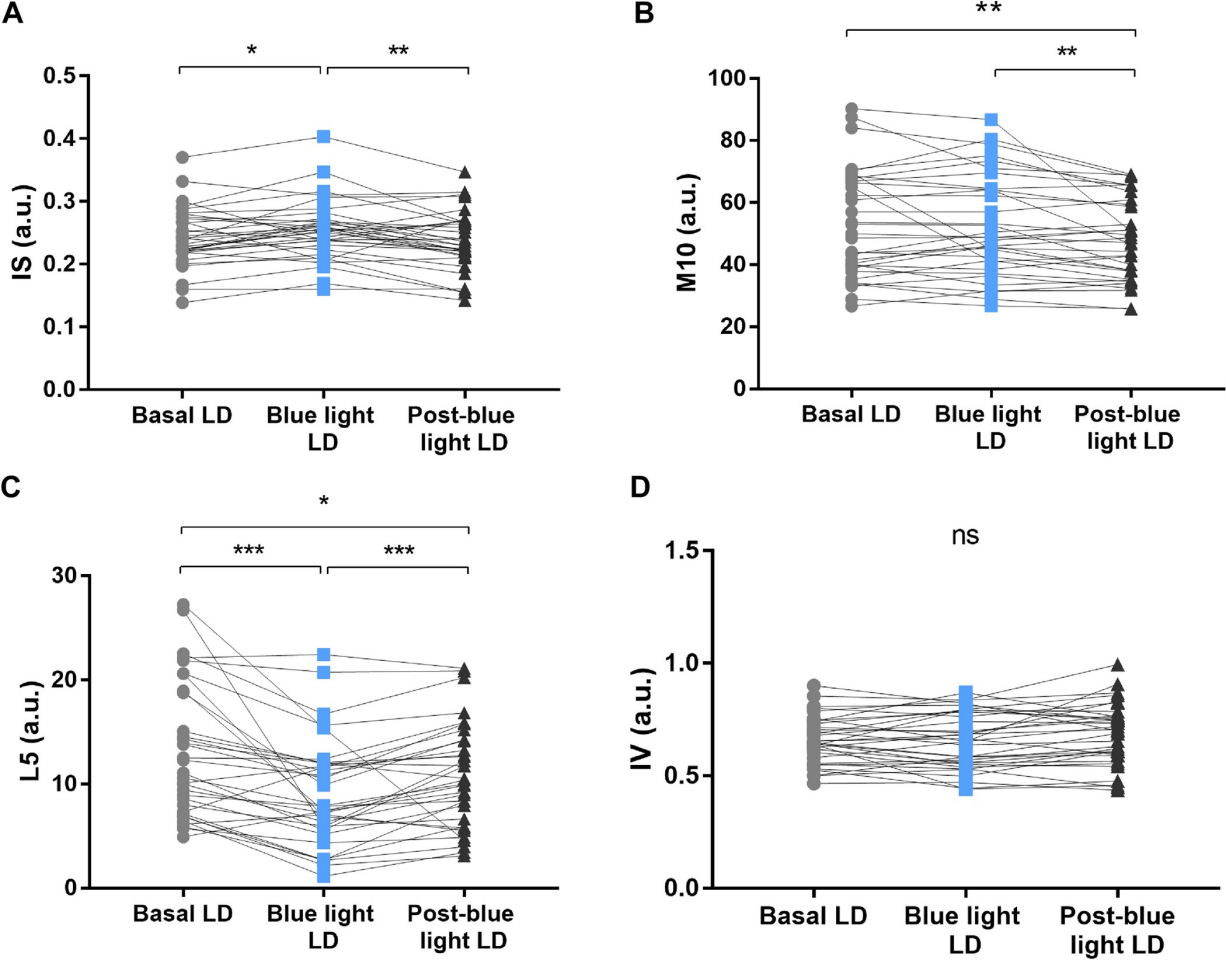
Non-parametric variables. Descriptive graphs of the IS (A), M10 (B), L5 (C), and IV (D) of aged rats (n = 33) locomotor activity submitted to the Basal LD (for 14 days), Blue light LD (for 14 days), and Post-blue light LD (for 14 days) conditions. Values expressed as median ± interquartile range. Circles, squares, and triangles represent values of each animal evaluated. a.u., arbitrary unit; IS, interdaily stability; M10, most active ten-hour period; L5, least active five-hour period; IV, intradaily variability; LD, light:dark; Wilcoxon test, p-value: *p<0.05; **p<0.01; ***p<0.001; ns, not significant.

The non-parametric variables of aged rats can be observed in Fig 6. Regarding IS, significant differences were observed between Basal LD (0.2377 ± 0.2197–0.2739) vs Blue light LD (0.2526 ± 0.2268–0.2701; Wilcoxon test, W = 279, p = 0.01), where the aged animals showed greater robustness of locomotor activity after exposure to blue light (Fig 6A). However, the robustness is diminished when blue light is stopped in Blue light LD vs Post-blue light LD (0.2294 ± 0.2133–0.2675; Wilcoxon test, W = -315, p = 0.004). Comparing the values of Basal LD vs Post-blue light LD (Wilcoxon test, W = -129, p = 0.25) there were no relevant differences (Fig 6A).

The M10 and L5 nonparametric variables also reproduced differences in locomotor activity during active and rest phases, respectively. M10 showed no consecutive changes in the activity pattern between Basal LD (49.96 ± 39.36–67.32) vs Blue light LD (48.75 ± 39.71–64.32; Wilcoxon test, W = 1, p = 0.98; Fig 6B). Differences were reported in comparisons between Blue light LD vs Post-blue light LD (47.16 ± 36.51–60.13; Wilcoxon test, W = -323, p = 0.003), and Basal LD vs Post-blue light LD (Wilcoxon test, W = -297, p = 0.007), where M10 values have decreased (Fig 6B). L5 showed a decrease in values after the animals were exposed to light therapy, i.e., Basal LD (11.05 ± 7.233–18.85) vs Blue light LD (7.86 ± 5.725–11.99; Wilcoxon test, W = -467, p<0.0001; Fig 6C). L5 values increased after withdrawal of blue light exposure, i.e., Blue light LD vs Post-blue light LD (10.36 ± 6.278–14.2; Wilcoxon test, W = 395, p = 0.0002). However, even after light treatment, L5 values remained lower than baseline, i.e., Basal LD vs Post-blue light LD (Wilcoxon test, W = -251, p = 0.024; Fig 6C).

The IV showed no significant differences between all stages evaluated, Basal LD (0.647 ± 0.5665–0.737) vs Blue light LD (0.653 ± 0.5535–0.787; Wilcoxon test, W = -409, p = 0.23), Blue light LD vs Post-blue light LD (0.701 ± 0.5915–0.7655; Wilcoxon test, W = 205, p = 0.06), and Basal LD vs Post-blue light LD (Wilcoxon test, W = 149, p = 0.18; Fig 6D). Additional parameters were observed: Start of activity phase (ZT); End of activity phase (ZT), and Mesor (a.u.). See S1 Table for details.

## 4 Discussion

The present work is the first to report the effect of blue light exposure on circadian activity parameters in aged rats. We observed that aged rats submitted to blue light treatment showed significant changes in locomotor rhythmicity parameters. The same happened with young rats exposed to blue light justifying the strength of light and its influence on the central nervous system, particularly on the CTS [63]. Here we present our new approach to aging treatment using blue light wavelength, which is effective in treating mood disorders in humans without harming the ocular structures [53, 64].

Typically, aging has significant effects on circadian rhythms [16, 65]. In order to validate the effects of aging on robustness of circadian rhythm, we used a young rats’ group to obtain values for a stable rest-activity rhythm. Young rats exhibited robust locomotor activity during baseline recording. From there, it was possible to state that as shown in other studies, we observed that aged animals present increased rhythm fragmentation, reduced amplitude, and decreased total daily activity. These findings are consistent with the results already described in rats [66], mice [65, 67], and flies [27]. Aging-associated changes in other rhythmic processes, such as melatonin release, body temperature, and the sleep/wake cycle have also been demonstrated in humans [13, 17].

Nonetheless, blue light exposure modified young and aged rats’ circadian rhythm, corroborating our hypothesis the attenuation of the circadian dysfunction in aging by increasing rhythm robustness. The rest-activity rhythm was evaluated from the actogram and the cosinor curve fitting method, describing the amplitude, mesor and acrophase of the locomotor activity [57]. However, as the rest-activity rhythm does not perform like a cosine function [62], other non-parametric variables were needed as IS, IV, L5, and M10 to see locomotor activity in detail [60]. Although M10 and L5 are based on rest-activity rhythm in humans, the use of these parameters in animals has been described in the literature in rats [68], mice [69], and marmosets [70, 71].

Exposure to blue light considerably changed the 24-hour activity profile observed in both aged and young rats. Important changes in the %AUC corroborate the direct effect of this photic exposure on locomotor activity [51]. In young rats, blue light was able to consolidate rest in ZT 0–6, increase activity in ZT 6–12 and ZT 12–18, and finally, decrease activity in ZT 18–24. In aged rats, the activity profile underwent changes similar to young ones. However, in ZT 12–18 in the Post-blue light LD stage it is possible to see a decrease in the %AUC compared to Blue light LD, which ends up with no significant difference compared to Basal LD. Blue light seems to have a better effect on the light:dark and dark:light transition phases (i.e., ZT 12–18 and ZT 0–6). This reaffirms the ideal moment for applying blue light is during the first half of the light phase, taking into account that application during the dark phase has been described as detrimental to memory and cognition in adult rats [72]. Furthermore, there is an alert about the effect of blue light on changes in the 24-hour activity profile, which seems to be directly related to the non-visual effects of light [51].

The blue light promoted changes in the cosinor parameters in young and aged animals. In aged rats, the amplitude of the locomotor activity increased during the application of blue light, but after when it was discontinued, the amplitude values dropped. In young rats, blue light had no effect on locomotor activity amplitude values. Regarding the acrophase, the aged animals showed a phase advance during the application of blue light, and after blue light removal showed a phase delay, both compared to baseline acrophase in the aged rats. In young rats, it was also possible to observe that this phase advance. Importantly, it is well established that light pulses applied in different phases of the day are able to promote advances or delay rhythmic variables [73]. Through this phase-dependent response curve, it is possible to explain how the CTS responds to light stimulation, synchronizing to the light:dark cycle [73]. Blue light changed the phase of entrainment of the locomotor activity by advancing the acrophase of all animals due to an immediate response to the light pulse emitted at the beginning of the light phase. This effect is not maintained throughout the stages, indicating the dependence on the presence of daily blue light to the maintenance of rhythmic improvement, which may be linked to the repeated stimulation of melanopsin during blue light-exposure [74].

Here, blue light appears to have a peculiar effect on total daily locomotor activity. Unlike the study by Wang et al. [75], in which a mouse model of Huntington’s disease treated with blue light showed an increase in total daily locomotor activity, in our study aged rats under blue light therapy showed a decrease in total daily locomotor activity after removal of blue light. In young animals, however, blue light decreased total daily locomotor activity. It is not clear why this happens, but it seems to be a peculiar behavior of blue light in which, in general, it decreases locomotor activity in the active phase of animals. Further investigation of these specific features in total daily locomotor activity is needed.

Variables related to rhythm stability, robustness, and fragmentation are usually measured to assess the efficiency of activity and rest in isolation [55, 59, 76]. These variables, in humans, are used in studies that analyze the effect of age, diseases and their respective treatments on circadian rhythm [28, 62, 77]. However, studies with animal models have been using these same variables to assess the rest-activity rhythm in the aged, such as studies with a non-human primate, Microcebus murinus [78–80]. In the present study, the robustness of the locomotor activity of aged rats was observed from the values of IS, which after exposure to blue light between the stages of Basal LD vs Blue light LD, increased the power of the rhythm of the aged animals. This means that aged animals treated with blue light show more robust locomotor activity and present better synchronization to external ZT of ~24 h [81]. In our study, no differences were observed between Basal LD vs Post-blue light LD, reinforcing the idea of the presence of light as an repeated exposure effect. These results are in line with investigations that postulate that blue light increases the robustness of circadian activity, strongly proposed by Wang et al. [75], Nagai et al. [82], and Elsabagh et al. [83]. This was also reinforced by the increase in circadian robustness found in young animals treated with blue light in our study. In young rats, IS values increased significantly, proving that blue light is capable of increasing rhythm strength even in a healthy and stable circadian rhythmicity.

Regarding the fragmentation of the rest-activity rhythm in aged animals, it is worth noting that for IV, high values indicate the presence of activity bouts in the rest phase and/or rest in the activity phase, which is a characteristic of aging [62]. In the present study, changes in the IV after exposure to blue light were not significant. It is worth mentioning that the blue light did not modify the IV neither in young rats nor in old rats. Our results are similar to those reported by Wang et al. [75], in mouse model of HD, in which no change of IV were obtained after blue light exposure. Fragmentation, usually related to disturbances during the sleep phase, was observed in our study. This is largely due to the reduced aging plasticity of the SCN clock [65]. However, why the blue light did not modify the IV in young rats remains unanswered. Here again we have a peculiarity of exposure to blue light.

The L5 variable, which measures diurnal activity in rodents and nocturnal activity in humans, corresponds to the ability to maintain a consolidated rest [62]. Increased L5 values mean the presence of activity in the resting phase, indicating the sleep is fragmented by awakenings [76]. Instability of the rest-activity rhythm in aged individuals, evidenced by an increase in L5, has been reported in humans [28, 59, 77] and in non-human primates [70, 71, 79, 80]. Our results show that during exposure to blue light, aged rats had lower L5 values, suggesting a more consolidated resting phase with less movement. The same happened with young rats, as blue light also promoted a decrease in L5 values, further improving rest for these animals. This shows, as postulated in the literature, that blue light is a possible agent in consolidating the sleep phase [63]. Furthermore, the effect after the removal of blue light in the aged rats was not immediate, as L5 values remained lower than in the baseline. Corroborating the 24-hour activity profile, blue light was efficient in consolidating the sleep phase, especially in the first half of the light phase (ZT 0–6).

During sleep-wake, the intensity of activity is multifactorial. The ability of the CTS to concentrate activity in one phase, the cortical activation by the midbrain, and the integrity of the motor system are examples of this ability [71, 72, 84]. High M10 values indicate predominance of locomotor activity [81], which was not observed in our results. In young animals, blue light did not promote significant changes in M10 values. In aged animals, when comparing the stages Basal LD vs Blue light LD, blue light also had no direct effect on the change in M10 values. However, in the Post-blue light LD stage, it was possible to observe that the M10 values reduced considerably. As in total daily locomotor activity, M10 seems to behave in the same way. In both cases, we have an interesting finding: blue light, despite not increasing M10 values, does not accelerate the reduction of these values. Light once again shows an effect of dependence on the presence of daily blue light for rhythmic maintenance [51]. Thus, deeper investigation should be carried out to better understand M10 reduction and its causes.

The CTS is known to modulate physiological and behavioral rhythms, such as patterns of activity and rest, alertness, hunger and appetite, and glucose and insulin sensitivity [81, 85]. Disturbances in the expression of these rhythms have been linked to health problems. For instance, in models of Parkinson’s disease [86, 87], Huntington’s disease [75, 88], and Alzheimer’s disease [89], blue light has been used as a treatment to reduce or delay the symptoms of neurodegeneration. Despite the growing body of evidence in the field, further and more in-depth studies with animal models and the evaluation of rest-activity rhythm parameters are necessary to understand the effects of blue light on circadian rhythmicity and its neuronal framework, particularly on the neuropeptidergic content of the SCN. Studies are needed to assess how blue light modulates neural components and in which way it occurs.

Aging is associated with a decline in sensory capacities, including visual ones [90, 91]. Age-related changes in the retina include loss of photoreceptors [92], rod bipolar cells [93], retinal ganglion cells [94], and retinal pigmented epithelial cells [95]. Age-related loss of photoreceptors may be greater in albino rats when compared to pigmented rats, due to greater susceptibility to light-induced damage [91]. Another explanation for the decline in retinal sensitivity with aging is that aged rats have increased lens opacities. Studies have shown that albino mice are more likely to develop significant lens changes than pigmented mice at 12 and 24 months of age [96]. As described by Tosini et al. [53], blue light is necessary in these cases of damage to the photoreceptors, especially in albino rats, as used in the present study. Exposure to blue light at ~150 lux was proven capable of promoting changes in rhythmic robustness in the rats evaluated in this study, preserving retinal plasticity. It is important to highlight that the results described in this study were in animals with albino eyes, and the particularities between albino and pigmented eyes must be taken into account, for example, the different regulation of expression and amount of melanopsin in pigmented eyes [91]. But we reinforce that if these effects were found in albino animals, in other animals with pigmented retina where the action of melanopsinergic activity is also significant, we believe that the effects can be significant as well.

In mammals, as a consequence of the expression of the melanopsin protein, ipRGCs receive photic signals through conventional photoreceptors and external photic stimuli [50]. This can happen even in cases of absence of main photoreceptors such as cones and rods [74]. The ipRGCs are key players in transmitting blue light information to multiple brain regions, such as the SCN, regulating non-visual brain functions [63]. It is worth noting that ipRGCs are conserved structures in the retina of several mammalian species, including humans [63]. The key role of melanopsin in the non-visual pathway is described, such as pupillary response to light, photoentrainment, photophobia and negative masking of locomotor activity [97]. One of the possible explanations for the improvement dependent on repeated exposure to blue light would be melatonin suppression and negative masking, both of which have been described in previous behavioral studies of light exposure [98, 99]. Findings like these strongly contribute to melanopsin as a photoentrainment agent, however it is not possible to say that blue light is masking the rest-activity rhythm of aged rats without evaluating the brain neuronal structures linked to the CTS.

One of the limitations of the present study is that we could not completely exclude the effects of senility in our experimental animals. Another limitation is that we cannot rule out the possibility that the improvement in locomotor behavior happened in an exposure-dependent manner due to the short exposure/therapy time that the rats underwent. Perhaps an increase in the temporal window of exposure can feed back the central nervous system and promote perennial changes to the CTS. Studies approaching longer light-exposure are still extremely important for possible long-term effects on the subjects investigated. More pre-clinical works are needed to optimize the treatment strategies as well as develop an understanding of the underlying mechanisms, as dose-response and more longitudinal analyses.

In addition, our results seem to contribute to use of blue light phototherapy as a productively effective chronotherapeutic method. In a translational perspective, blue light treatment can be a complementary and non-invasive method to soften the deleterious effects of aged CTS in humans, which is already being used effectively in neurodegenerative diseases and mood disorders [45, 52, 100, 101]. Blue light chronotherapy could be used concomitantly with drugs in cases of neurodegeneration to alleviate circadian rhythm disturbances, with easiness of applicability in hospitalized patients, home care, nursing homes, institutions for the aged, clinics, and others. It is worth remembering that phototherapy can also be associated with other chronotherapies, such as physical activity and temporal food restriction [102].

In conclusion, exposure to blue light during the first half of the light phase appears to have positive effects on the robustness of the circadian rhythm of locomotor activity in aged rats, particularly increasing the amplitude, increasing the interdaily stability, promoting a phase advance of acrophase, and consolidation of the resting phase. In young rats, blue light was also able to promote a considerable increase in circadian robustness. Finally, blue light seems to have a better effect on the light:dark and dark-light transition phases (i.e., ZT 0–6 and ZT 12–18).

## Supporting information

S1 Table Locomotor activity of aged animals (n = 33) exposed to the baseline (for 14 days), treatment (for 14 days), and post-light treatment (for 14 days) stages. Values expressed as median ± interquartile range.
(DOCX)
