# Supplementary material for: Blue light exposure-dependent improvement in robustness of circadian rest-activity rhythm in aged rats
Source: PLoS One. 2023 Oct 4;18(10):e0292342. doi: 10.1371/journal.pone.0292342 (PMC10550138; doi:10.1371/journal.pone.0292342)
Supplement: S1 Table — Values expressed as median ± interquartile range. (DOCX) [file pone.0292342.s001.docx]

**Supporting information**

**S1 Table.** **Locomotor activity of aged animals (n=33) exposed to the baseline (for 14 days), treatment (for 14 days), and post-light treatment (for 14 days) stages.** Values expressed as median ± interquartile range.

| Parameters | Aged (16mo) | | |
| --- | --- | --- | --- |
|  | Basal LD | Blue light LD | Post-blue light LD |
| Start of activity phase (ZT) | 12.05 ± 11.61-12.41 | 11.71 ± 11.3-12.02^*^ | 11.95 ± 11.7-12.34^#^ |
| End of activity phase (ZT) | 23.07 ± 22.56-23.36 | 22.99 ± 22.43-23.34 | 23.19 ± 22.86-23.39^#^ |
| Mesor (a.u.) | 30.5 ± 22.57-42.77 | 30.17 ± 22.32-41.36 | 29.13 ± 21.49-40.06^*#^ |

Wilcoxon test; p-value: p<0.05; * indicates significant difference compared to Basal LD. # indicates significant difference compared to Blue Light LD; a.u., arbitrary unit; ZT, zeitgeber time; LD, light:dark.
